# Solution casting of chitosan membranes for *in vitro* evaluation of bioactivity

**DOI:** 10.1186/1480-9222-15-11

**Published:** 2013-11-06

**Authors:** Ramona Lieder, Mariam Darai, Gissur Örlygsson, Olafur E Sigurjonsson

**Affiliations:** 1 REModeL Laboratory, The Blood Bank, Landspitali University Hospital, Snorrabraut 60, 105 Reykjavik, Iceland; 2 School of Science and Engineering, Reykjavik University, Menntavegur 1, 101 Reykjavik, Iceland; 3 Innovation Center Iceland, Arleynir 2-8, 112 Reykjavik, Iceland; 4 Biomedical Center, University of Iceland, Vatnsmyrarvegur 16, 101 Reykjavik, Iceland

**Keywords:** Chitosan, Membranes, Characterization, Titanium, Chitosan derivatives, MC3T3-E1, Fibronectin adsorption, Crosslinking

## Abstract

**Background:**

Considerable research is focusing on the surface modification of titanium implants for the treatment of orthopaedic tissue injuries to increase the success of orthopaedic fixations. Chitosan is one of the natural materials under investigation based on several favourable properties. Numerous techniques have been described for the preparation of chitosan membranes, including solution casting methods for the investigation of bioactivity before applying coatings onto potential titanium implants. Solution casting enables the easy in-house evaluation of chitosan membranes and allows for the selection of promising chitosan materials.

**Results:**

We present a method for the standardized and easily applied preparation of chitosan membranes by solution casting. This protocol is suitable for chitosan materials spanning a wide degree of deacetylation, being derived from different chitin sources and chitosan derivatives with novel properties. We detail the preparation and quality control methods in order to prepare membranes with favourable bioactivity, sustaining cell attachment and proliferation for extended culture periods.

**Conclusions:**

The possibilities associated with the use of chitosan in tissue engineering applications are far from being exhausted and numerous challenges remain prior to successful translation into the clinics. Based on our experience, we have developed simple in-house methods for quality control of homogeneous membrane casting and early prediction of successful experimental outcome.

## Background

Titanium implants for the treatment of orthopaedic tissue injuries are recommended for a number of load-bearing applications, but still lack improvement at the bone-biomaterial interface [1,2]. Research focusing on the surface modification of these materials could considerably increase the success of orthopaedic fixations. Chitosan, the partly deacetylated configuration of chitin, is one of the natural materials under investigation to improve implant integration and cellular attachment [3-5]. Several promising properties are attributed to chitosan, including biocompatibility, non-toxicity and *in vivo* degradation [6]. Based on the chemical nature of chitosan, negatively charged cytokines and growth factors can be retained at its surface and exert favourable effects on osteogenesis *in vitro* and *in vivo*[7,8]. The straight-forward use of chitosan and the easy molding abilities have long been recognized, and make this polymer an attractive tool for tissue engineering and regenerative medicine applications [9,10].

Several properties of chitosan have been reported to strongly influence cell attachment and bioactivity *in vitro*, including the degree of deacetylation (DD), origin of the chitin source and the surface characteristics of the final membrane/coating [11,12]. The effect on cellular behaviour was shown to be cell-type specific, but generally a lower degree of deacetylation is thought to induce healing without scar tissue formation, whereas higher degrees of deacetylation are more beneficial for cell attachment and proliferation [13,14].

Numerous techniques have been developed for the preparation of chitosan membranes, including solution casting, layer-by-layer self-assembly and silanization methods for bonding to titanium implants [3,15,16]. Solution casting of chitosan membranes on tissue culture plastic is a widely used method for the *in vitro* evaluation of bioactivity before applying coatings onto potential titanium implants. This technique provides an easy in-house investigation of cell attachment following standard laboratory protocols and allows for the selection of promising chitosan materials in accordance to general requirements for coated implants in tissue engineering applications. However, there are a vast number of protocols available for solution casting of chitosan membranes, often restricted to the use of a specific degree of deacetylation [1,8,13,17]. Additionally, cell attachment and proliferation are frequently significantly lower than on traditionally used tissue culture plastic and cannot be sustained for extended periods of time [1].

We have recently developed a standardized and easily applied protocol for the solution casting of chitosan membranes spanning a wide degree of deacetylation, displaying favourable bioactivity by sustaining cell attachment and proliferation for extended culture time [18]. This protocol is suitable for the use of chitosan materials derived from varying chitin sources and the investigation of chitosan derivatives with modified properties.

## Results and discussion

**Figure 1:**
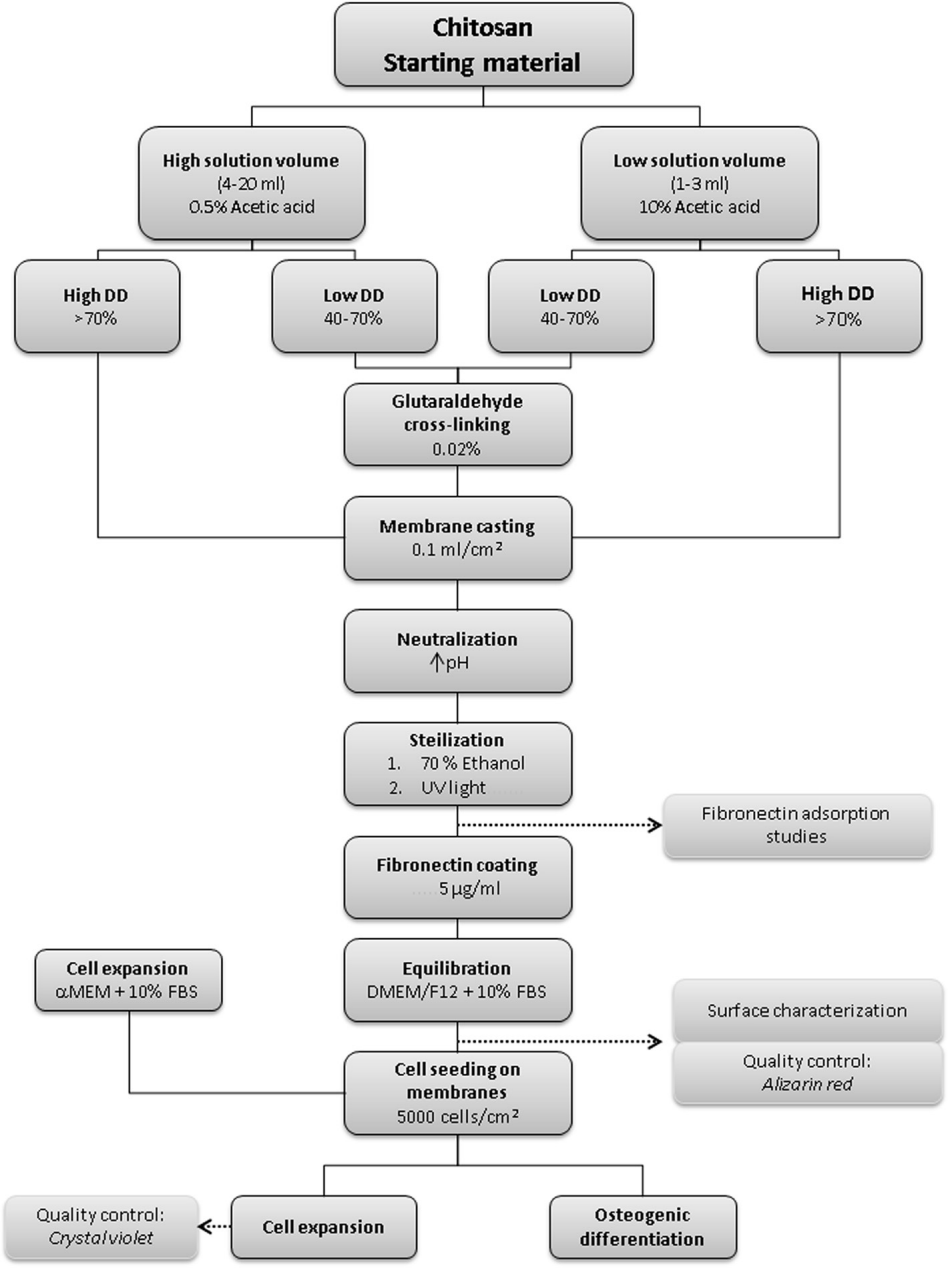
**Work flow chart for the solution casting of chitosan membranes.** The preparation scheme is visualized stepwise for the successful casting of chitosan membranes on tissue culture plastic. Additional analysis and quality control are indicated where appropriate.

The preparation of chitosan membranes using solution casting methods follows a three day procedure. For best results, the procedure should be performed without stopping points in order to ensure best bioactivity results. However, after initial sterilization on day 3, chitosan membranes can be stored for several weeks until use in experiments or analysis of surface characteristics. The preparation of chitosan membranes can be performed on the bench. An overview of the work flow required for membrane preparation is shown in Figure 1. We have found that the preparation of chitosan solution for membrane casting should follow slightly different procedures depending on the volume needed.^b^ For optimal thickness of chitosan membranes, 0.1 ml of chitosan solution are cast per cm^2^ of tissue culture plastic. At least 10% extra should be prepared to account for losses during the preparation. Both chitosan powder and chitosan flakes with different degree of deacetylation (DD) can be used in this protocol. However, chitosan flakes are generally more difficult to handle than chitosan powders. Depending on the molecular weight of chitosan, the solutions will differ in viscosity, with less viscous solutions being easier to process. The protocol can be easily scaled to the volume required, and is here described for “high volume” (*See* Subsection High volume (4-20 ml) and “low volume” (*See* Subsection Low volume (1–3 ml).) set-up. Solution casting methods are described for the coating of 6-well plates. Respective volumes needed for smaller sized tissue culture plastic are described in Table 1.

**Table 1:** Volume for preparation of chitosan membranes dependnt on the type of culture plate

	**Chitosan solution**	**NaOH (neutralization)**	**70% ethanol (sterilization)**	**Fibronectin solution**	**Cell culture media**	**Alizarin Red Stain**	**Crystal Violet Stain**
**6-well plate**	1 ml	2 ml	2 ml	2 ml	3 ml	2 ml	2 ml
**12-well plate**	500 μl	1 ml	1 ml	1 ml	1.5 ml	1 ml	1 ml
**24-well plate**	250 μl	500 μl	500 μl	500 μl	1 ml	1 ml	1 ml
**96-well plate**	100 μl	200 μl	200 μl	200 μl	100 μl	100 μl	100 μl
**2-well slide chamber**	500 μl	1 ml	1 ml	1 ml	2 ml	1 ml	1 ml

Proper casting of the chitosan membranes can be inspected by visual examination of the plastic ware against a light source. Small areas of in-homogenous casting then become visible. Since Acetic Acid is used to decrease the pH of the chitosan solution and enable solubility, chitosan membranes need to be neutralized after the drying process to render them water insoluble.

In order to not compromise cell culture studies, chitosan membranes follow a dual sterilization procedure, using both 70% ethanol and UV-light. After sterilization, all work with chitosan coated plastic ware should be performed in a sterile fume hood. Coating with the adhesion protein fibronectin is used to promote initial cell attachment to the chitosan surface and is therefore essential for the success of the *in vitro* cultures.

**Figure 2:**
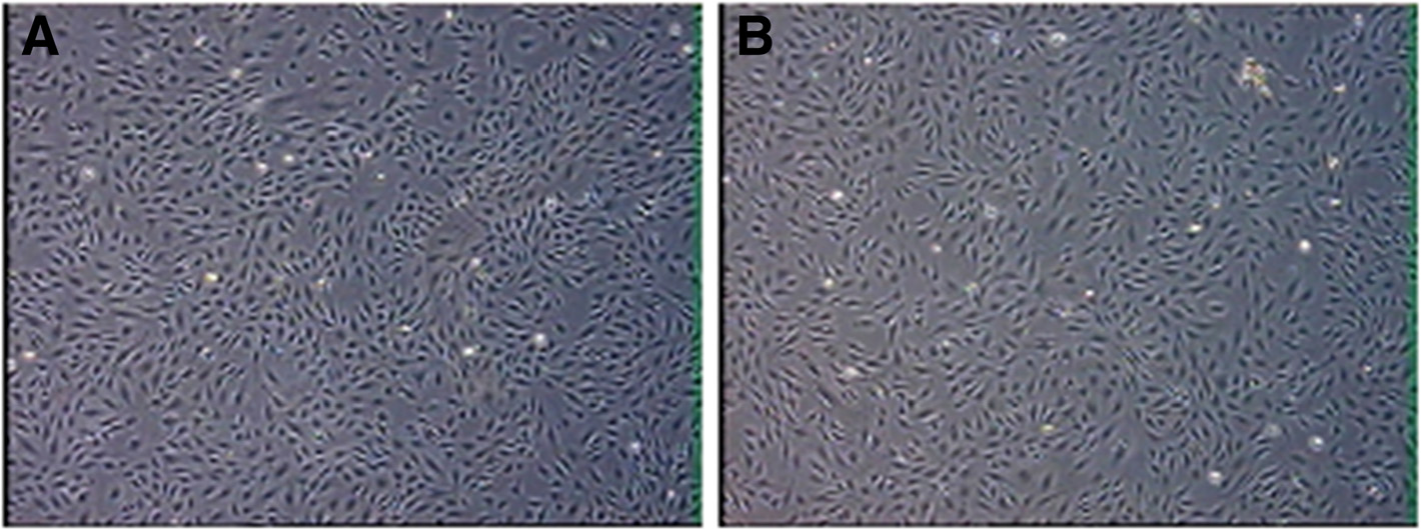
**Images of MC3T3-E1 pre-osteoblastic cells grown on chitosan membranes prepared from different chitin sources. A)** Is an image of cells after 48 h grown on crab shell derived chitosan with 87% degree of deacetylation. **B)** Is an image of cells after 48 h grown on shrimp shell derived chitosan with 87% degree of deacetylation. Images were taken in an inverted microscope.

**Figure 3:**
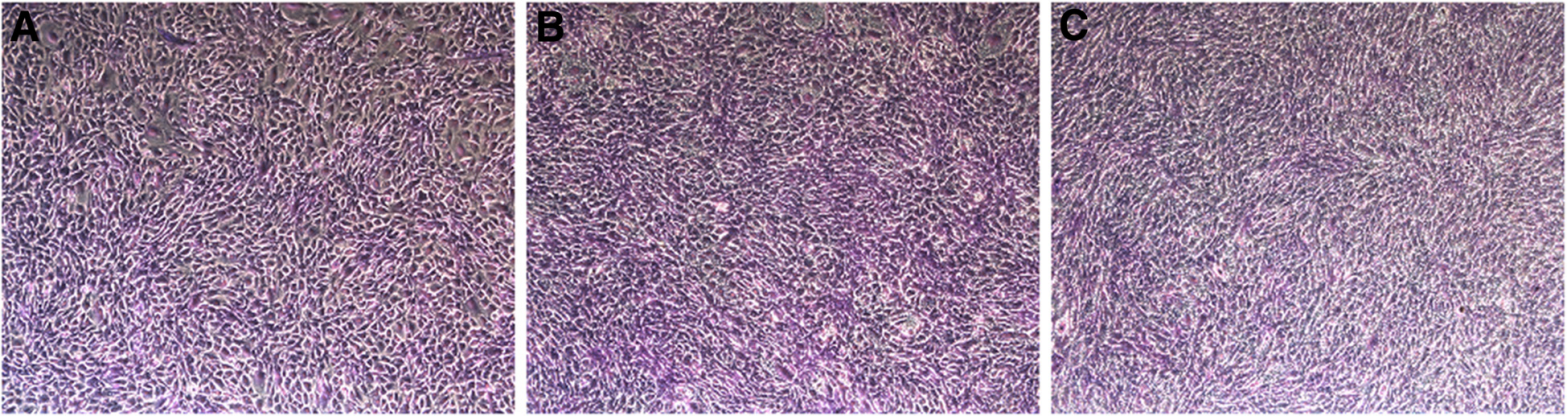
**Images of MC3T3-E1 pre-osteoblastic cells grown on chitosan membranes prepared from chitosan derivatives. A)** Is an image of cells grown on a chitosan free base derivative for 7 days. **B)** Is an image of cells grown on Chitosan p-Toluensulfonic acid-salt (PTSA salt) for 7 days. **C)** Is an image of cells grown on Chitosan-Bromide salt for 7 days. Cells were stained with crystal violet and images were taken in an inverted microscope.

The mouse pre-osteoblastic cell line MC3T3-E1 can be grown on chitosan membranes in an undifferentiated state using basic growth media or induced to undergo osteogenic differentiation. Cell attachment can be maintained for up to 24 days under differentiation conditions. Generally, it is sufficient to set-up MC3T3-E1 cultures two days before initiation of the chitosan casting protocol, at a density of 3,500 cells/cm^2^. In our experience, subclone 4 of this particular cell line is best suited for induction of osteogenic differentiation. Representative images of cells grown on different sources of chitin and chitosan derivatives used for chitosan membrane casting are shown in Figure 2 and Figure 3. Please refer to reference 18 for more representative images.

The lower the DD, the more soluble chitosan membranes generally become based on the degradation of secondary structures [19]. From our experience with membranes prepared from a low DD (40-70%), cross-linking with glutaraldehyde is required to avoid dissolution during long term cultures. Membranes prepared from low DD materials will only be stable for one week in culture media before substantial dissolution can be observed. In order to avoid glutaraldehyde-related toxicity (“leaking of glutaraldehyde into the culture media”) and still provide sufficient stability, a fine balance concerning the amount of glutaraldehyde needs to be achieved. We have found that chitosan membranes with low DD that are internally cross-linked with 0.02% glutaraldehyde are stable for long-term cultures and still retain favourable bioactivity and cell attachment.

Bioactivity and cell attachment are not only dependent on the DD and the origin of the chitin source, but are strongly influenced by surface characteristics, water contact angles and the ability of chitosan membranes to retain fibronectin [11,12,20,21]. Fibronectin adsorption can be examined by simple in-house ELISA and measured in a spectrophotometer at 400 nm. This protocol has been adapted from Uygun et al. [22] and modified appropriately. Water contact angle measurements are best performed on microscopy slides to avoid unnecessary manipulation of the specimen. We have found that 2-well chamber slides from LabTek are well suited for solution casting of chitosan membranes, providing easy handling and the appropriate tissue culture plastic surface. In order to determine the average surface roughness and topography, chitosan membranes can be prepared in 12-well plates. This decreases the amount of sample needed and still enables successful analysis after cutting the plastic sides off the well. Since the area studied during Atomic Force Microscopy is very small, the cutting does not affect the surface characteristics in the middle of the well.

**Figure 4:**
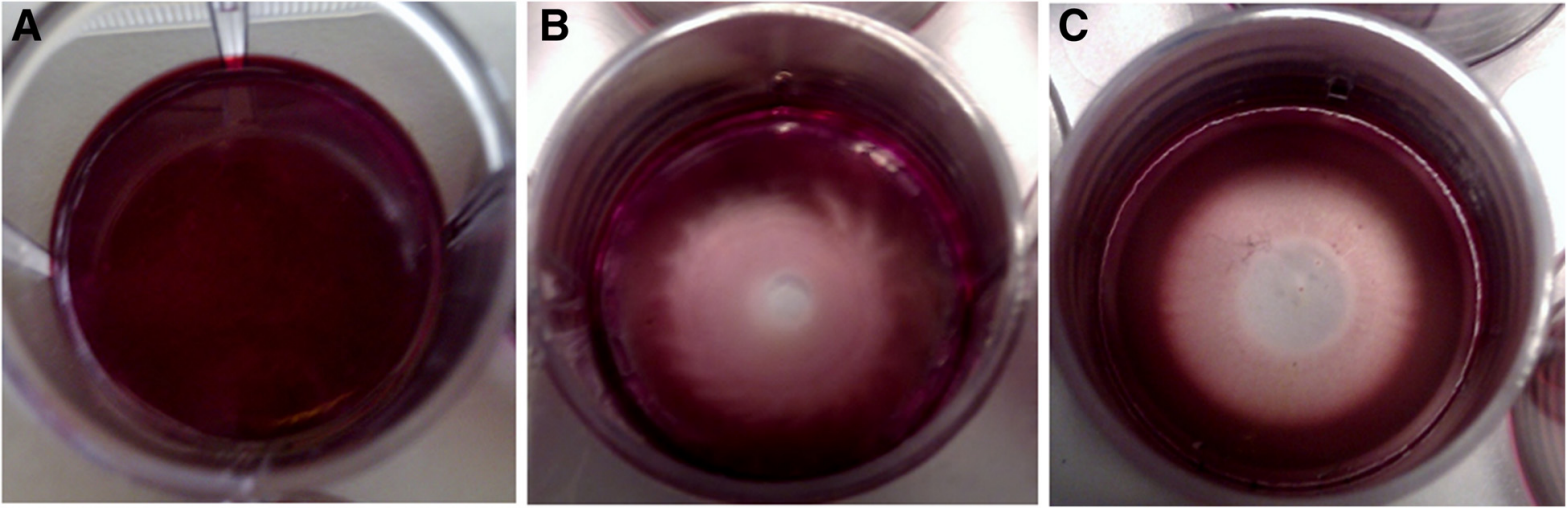
**Chitosan membranes stained with Alizarin Red Stain for comparison of homogeneous and in-homogenous membrane casting. A)** Is an image of a homogeneously distributed chitosan membrane prepared from crab shell chitosan with 87% degree of deacetylation. **B)** Is an image of an in-homogeneously distributed chitosan membrane prepared from modified crab shell chitosan with 87% degree of deacetylation. A spiral-like distribution of the membrane can be observed. **C)** Is an image of an in-homogeneously distributed chitosan membrane prepared from N-lauroyl chitosan derivatives with a degree of substitution of 0.05%.

The *in vitro* success of chitosan membranes used for cell attachment strongly depends on the homogeneity of the final membrane. This can be easily assessed using a modified Alizarin Red Staining protocol. Based on the chemical characteristics of chitosan, acidic dyes, including Alizarin Red S, are robustly retained. Thereby, defects in the membrane casting can be easily spotted. This method is also useful for the investigation of dissolution after extended culture periods on chitosan membranes. Representative images of homogeneously and in-homogeneously distributed chitosan membranes after staining with Alizarin Red are shown in Figure 4.

Cells grown on chitosan membranes can be easily visualized by standard Crystal Violet Staining. Based on the chemical characteristics of the Crystal Violet Dye, chitosan membranes will remain unstained. However, lipophilic chitosan derivates can react with the triphenyl methane structure, which will result in dark violet staining of chitosan membranes. In that case, cell bodies are best observed using an inverted microscope equipped with a camera.

## Conclusions

The possibilities associated with the use of chitosan in tissue engineering applications are far from being exhausted and numerous challenges remain prior to successful translation into the clinics. However, a vast body of conflicting literature is available describing the attachment, proliferation and osteogenic differentiation of osteo-progenitor cells grown on chitosan membranes with different degrees of deacetylation [23]. This is mainly due to the lack of consistency in providing sufficient data on molecular weight, source of chitosan and sample preparation in order to compare and draw conclusions [23]. The often scarcely detailed methodological sections in biomaterials-related publications strongly impede reproducibility.

Here, we describe a protocol for the solution casting of chitosan membranes that is suitable for the use of chitosan from different chitin sources, chitosan with a wide range of degree of deacetylation and chitosan derivatives with novel properties. We provide a step-by-step procedure that results in cellular attachment comparable to tissue culture plastic controls and allows for the maintenance of cultures for extended periods of time. Based on our experience, we have developed simple in-house methods for quality control of homogeneous membrane casting and early prediction of successful experimental outcome.

## Methods and materials

### Solution casting of chitosan membranes

A standard protocol was developed for the solution casting of chitosan membranes. Briefly, a 1% (w/w) chitosan solution in deionized H_2_O with 50 mM glacial acetic acid (100%) was stirred on a magnetic stirrer until fully dissolved. Homogeneous membrane casting and removal of undissolved particles was asserted by 1 h centrifugation at 5,000 rpm before solution casting. A total of 0.1 ml chitosan solution/cm^2^ was cast into tissue culture-treated plates and dried over-night in an incubator at 37°C. Next, chitosan membranes were neutralized with 0.1 M NaOH, sterilized with 70% ethanol, and further sterilized under UV-light. To improve attachment, membranes were incubated with a 5 μg/ml fibronectin solution in sterile PBS for 3 h at 37°C. Equilibration was performed in Dulbecco’s modified eagle medium (DMEM/F12) media supplemented with penicillin/streptomycin and 10% heat-inactivated fetal bovine serum (FBS).

To improve stability of low DD chitosan membranes during long-term cultures, respective membranes were internally cross-linked using 0.02% glutaraldehyde solution.

### MC3T3-E1 cell seeding and culture

MC3T3-E1 cells were maintained in α-MEM media supplemented with penicillin/streptomycin and 10% heat-inactivated fetal bovine serum. During experiments on chitosan membranes, expansion media was replaced by DMEM/F12 media supplemented with penicillin/streptomycin and 10% heat-inactivated fetal bovine serum to enhance attachment and proliferation.

### Surface characterization

Water contact angles were determined using a KSV CAM200 optical contact angle meter (KSV Instruments) and a droplet volume of 5 μl distilled water. The contact angle measurement was started 10 s after drop down and calculated using the Laplace & Young equation. Measurements were performed at room temperature and ambient humidity.

Surface topography of chitosan membranes was evaluated using an XE-100 atomic force microscope (Park Systems) with a scan size of 5 nm and a scan rate of 0.15 Hz in noncontact mode.

For fibronectin adsorption studies, chitosan membranes and tissue culture plastic controls were incubated with a 5 μg/ml fibronectin solution in PBS over night at 4°C. Samples were then washed four times with PBS and nonspecific adsorption was blocked with 1% BSA in PBS for 2 h. After washing three times with PBS, samples were incubated with rabbit anti-fibronectin antibody for 2 h. Samples were then washed three times with PBS and incubated with goat anti-rabbit IgG Alkaline Phosphatase-conjugated antibody for 2 h. Samples were then washed three times with PBS and incubated with the substrate p-nitrophenyl phosphate for 30 min. Optical density was measured at 405 nm in a MultiSkan spectrometer (Thermo Scientific).

### Quality control

Cells were periodically checked in an inverted microscope for phenotype consistency and morphology. 0.5% crystal violet solution in methanol was used to stain cells and images were taken in an inverted microscope with IC Capture 2.0 Software.

To determine homogeneity of membrane casting and dissolution after prolonged culture in aqueous media, membranes were washed three times with dH_2_O and then stained with a 2% Alizarin Red solution at pH 4.2 for 5 min. Then, membranes were washed four times with dH_2_O, dried upside down over night and then images taken in an inverted microscope with IC Capture 2.0 Software.

### Materials

#### Solution casting of chitosan membranes

1. Chitosan powder or flakes (e.g. Chitosan from crab shells; Cat. No. 50494, Sigma)

2. 100% Glacial Acetic Acid (Merck)

3. Transfer pipettes (Cat. No. 86.1172.010, Sarstedt)

4. Glass test tubes (Sigma)

5. 15 ml plastic conical tubes (Falcon)

6. Centrifuge with buckets for 15 ml tubes (5,000 rpm acceleration)

7. Flat bottom plates with low evaporation lid; tissue culture treated by vacuum gas plasma (Falcon).^a^

8. Incubator heated at 37°C, no CO_2_ injection, no humidity control

9. Sodium hydroxide pellets (Cat. No. 6482.5000, Merck). Dissolve pellets in water to obtain 0.5 M solution.

10. 96% ethanol. Dilute in dH_2_O to obtain 70% ethanol.

11. Fibronectin from Human plasma (Cat. No. 356008, BD Biosciences). Aliquot (100 μl/ eppendorf) and store frozen at −20°C.

#### MC3T3-E1 culture on chitosan membranes

1. Mouse pre-osteoblastic cell line MC3T3-E1 (subclone 4; Cat. No. ATCC-CRL-2593, ATCC)

2. Dulbecco’s Modified Eagle Medium: Nutrient Mixture F-12 (DMEM/F12 1:1) with GlutaMAX (Cat. No. 31331, Gibco)

3. Minimum Essential medium alpha (αMEM) without ascorbic acid (Cat. No. A10490, Gibco)

4. Heat-inactivated fetal bovine serum (FBS; Cat. No. 10500, Gibco). Aliquot and store frozen at −20°C.

5. Antibiotics: Penicillin-streptomycin- glutamine mix (100× solution; Cat. No. 10378, Gibco). Aliquot and store frozen at −20°C.

6. β-glycerophosphate disodium salt hydrate (Cat. No. G9422, Sigma)

7. L-Ascorbic acid (Cat. No. A4403, Sigma)

8. 75 cm^2^ cell culture flasks (Nunc)

9. 2-well chamber slides with cover (Cat. No. 177429, Lab-Tek)

10. Cell incubator set at 95% humidity, 37°C and 5% CO_2_ in air

#### Materials needed for quality control

1. Glutaraldehyde Solution Grade I 50% (Cat. No. G7651, Sigma Aldrich). Store frozen at −20°C and thaw in the dark.

2. Alizarin Red S powder (Cat. No. A5533, Sigma Aldrich)

3. Ammonium hydroxide (Merck). Prepare 0.5% solution.

4. Crystal Violet powder (Cat. No. C3886, Sigma Aldrich)

5. Phosphate buffered saline (1× PBS; pH 7.2; Cat. No. 10010–015, Gibco)

6. Bovine Serum Albumin (BSA; Cat. No. A-4503, Sigma)

7. Rabbit anti-fibronectin antibody (Cat. No. F3648, Sigma Aldrich)

8. Goat anti-rabbit IgG Alkaline Phosphatase conjugated antibody (Cat. No. A9919, Sigma Aldrich)

9. Sigma FAST p-Nitrophenyl phosphate tablets (1 mg/ml p-Nitrophenyl phosphate and 0.2 M Tris buffer; Cat. No. N2770, Sigma)

### Step-wise protocol

#### Solution casting of chitosan membranes - Day 1

##### High volume (4–20 ml)

1. Weigh in 10 mg of chitosan material per ml of final volume desired, in a small plastic cup (~50-100 ml flat bottom cup)

2. Add 0.985 g/ml of dH_2_O using a transfer pipette

3. Add 5 μl/ml of 100% Acetic Acid in a fume hood

4. Stir solution on a magnetic stirrer. This may take between 5 min to 1 h depending on the chitosan material. Cover the plastic cup with aluminium foil and assure that stirring is not too strong, to avoid splashing of the solution onto the sides of the cup.^c^

5. Transfer chitosan solution into 15 ml Falcon tubes.

6. Centrifuge at 5,000 rpm for 1 h to remove air bubbles and un-dissolved particles

7. (When working with chitosan materials below DD70 please skip to Section Glutaraldehyde cross-linking for glutaraldehyde cross-linking of membranes with low DD)

8. For coating a 6-well plate, carefully place 1 ml of Chitosan solution (using a 1 ml micropipette) into the middle of the well. Air bubbles should be avoided.

9. Spread chitosan solution to the corners of the well with a continuous smooth hand movement. Be sure to completely cover the well with solution.

10. Occasional bubbles in the solution after casting can be removed by using a needle or a small pipette tip. All bubbles need to be removed before drying the membranes.

11. Dry membranes over night in an incubator at 37°C without CO_2_ injection or humidity control. Plates need to be uncovered to allow evaporation.

##### Low volume (1–3 ml)

1. For 2 ml of Chitosan Solution, weigh in 10 mg/ml of Chitosan powder or flakes in a clear test tube.

2. Add 0.7 g/ml of dH_2_O using a transfer pipette.

3. Add 100 μl/ml of 100% Acetic Acid in a fume hood.^b^

4. Re-suspend chitosan solution using a transfer pipette to wash off any chitosan material from the test tube walls.

5. Cover test tube with aluminium foil.

6. Heat test tube in a water bath at 50°C for at least one hour or until dissolved.

7. Transfer chitosan solution into 15 ml Falcon tubes.

8. *See* Subsection High volume (4-20 ml) and follow Steps 6–10.

#### Neutralization of chitosan membranes – Day 2

1. Add ca. 0.2 ml of 0.5 M NaOH/cm^2^ well area to each well.

2. Incubate for 30 min on a shaker at room temperature. Shaking should be slow and solution should be just moving. To account for the increased concentration of acetic acid during “low volume” preparations, neutralization time needs to be increased to 4 h.

3. Invert plates to remove NaOH and tap on a piece of paper to remove excess liquid.

4. Wash three times with ca. 0.2 ml of dH_2_O/cm^2^ well area for 10 min on a shaker at room temperature.

5. Invert plates to remove dH_2_O and tap on a piece of paper to remove excess liquid.

6. Dry membranes over night in an incubator at 37°C, without CO_2_ injection or humidity control. Pates should be uncovered to allow for evaporation.

#### Sterilization and fibronectin coating of chitosan membranes – Day 3

##### Sterilization of chitosan membranes

1. Prepare 70% ethanol for disinfection.

2. Add ca. 0.2 ml of 70% ethanol/cm^2^ well area into each well and incubate 30 min at room temperature without shaking.

3. Invert plates to remove ethanol and tap on a piece of paper to remove excess liquid.

4. Dry membranes for 1 h in an incubator at 37°C without CO_2_ injection or humidity control. Plates should be uncovered to allow for evaporation.

5. At this point, chitosan coated plastic ware can be covered with parafilm for storage at 4°C in the dark until further use.

6. Place uncovered chitosan coated plastic ware under the UV-light lamp in a fume hood for 30 min.

7. (*See* Subsection Fibronectin adsorption studies).

##### Fibronectin coating of chitosan membranes

1. Prepare a 5 μg/ml Fibronectin solution in 1× PBS into each well, by diluting frozen 1 mg/ml fibronectin stock solution.

2. Add ca. 0.2 ml of Fibronectin solution/cm^2^ well area into each well and incubate on a shaker for 3 h at 37°C. Solution should be just moving on the shaker to allow fibronectin to attach to the surface.

3. Invert the plate to remove fibronectin solution and tap plastic ware on a paper to remove excess liquid.

4. (*See* Section Surface characterization of chitosan membranes).

5. Equilibrate chitosan membranes for 20 min in DMEM/F12 media supplemented with 10% FBS and penicillin/streptomycin. Culture media will turn pink due to the high pH of the fibronectin solution. Equilibration is needed both for removal of excess fibronectin and buffering of pH.

6. Discard equilibration media before cell seeding.

#### MC3T3-E1 cell seeding and culture – Day 3 - Day 24

1. Expand MC3T3-E1 cells in αMEM media supplemented with 10% FBS and streptomycin/penicillin until 80% confluent.

2. 5,000 cells/cm^2^ are required for seeding on chitosan coated plastic ware.

3. Trypsinize and count cells.

4. Re-suspend cells in DMEM/F12 media supplemented with 10% FBS and streptomycin/penicillin (basic expansion media).

5. Place 5,000 cells/cm^2^ into the well and cover with appropriate volume of basic expansion media (*see* Table 1).

6. Incubate cell cultures at 37°C, 95% humidity and 5% CO_2_.

7. Fully replace cell culture media every second day.^d^

8. To induce osteogenic differentiation of MC3T3-E1 cells, incubate cells for one night in basic expansion media to allow for initial attachment.

9. Osteogenic induction media is prepared from basic expansion media by addition of 2 mM β-Glycerophosphate and 50 μl/ml ascorbic acid. Mix thoroughly on a vortexer.

10. Remove basic expansion media and replace with osteogenic induction media. Again, culture media should be fully replaced every second day. First signs of osteogenic differentiation will be visible after 7–10 days (calcification, collagen type I deposition, cell contraction and change in phenotype).

#### Glutaraldehyde cross-linking

1. *See* Subsection High volume (4-20 ml) and Subsection Low volume (1–3 ml) for “high” and “low volume” preparation. Follow the procedure until Step 6.

2. During the centrifugation time (Step 6), thaw glutaraldehyde solution in the dark

3. Prepare an eppendorf tube containing 100 μl distilled water.^e^

4. Prepare a 0.02% glutaraldehyde solution (0.02% of the final volume of chitosan solution). Add into the eppendorf tube containing 100 μl distilled water. Mix well!

5. Transfer chitosan solution after centrifugation into a fresh 15 ml Falcon Tube.

6. Add the glutaraldehyde – distilled water solution to the chitosan solution.

7. Mix thoroughly on a vortexer.

8. Cast Membranes according to Step 8–10 in Subsection High Volume (4-20ml) 3.1.1.^f^

9. *See* Section Neutralization of chitosan membranes – Day 2 and Section Sterilization and fibronectin coating of chitosan membranes – Day 3 for neutralization and sterilization of cross-linked chitosan membranes

#### Surface characterization of chitosan membranes

##### Fibronectin adsorption studies

1. Prepare chitosan membranes in a 96-well plate following the procedure described in Section Solution casting of chitosan membranes - Day 1, Section Neutralization of chitosan membranes – Day 2 and Section Sterilization and fibronectin coating of chitosan membranes – Day 3 (Step 6).

2. Prepare a 5 μg/ml Fibronectin solution in 1x PBS, by diluting frozen 1 mg/ml fibronectin stock solution

3. Add 100 μl of fibronectin solution into each well and incubate over night at 4°C. Include non-coated tissue culture plastic as positive control for fibronectin adsorption.

4. Invert the plate to remove fibronectin solution and tap on paper to remove excess liquid.

5. Wash four times for 30 min with 200 μl 1x PBS at room temperature.

6. Tap plates on paper to remove excess liquid in-between washing steps.

7. Block unspecific adsorption by incubation with 1% BSA in 1x PBS for 2 h at room temperature.

8. Wash three times for 10 min with 200 μl 1x PBS at room temperature.

9. Tap plates on paper to remove excess liquid in-between washing steps.

10. Prepare a 1:15 000 dilution of primary antibody (rabbit anti-fibronectin antibody).

11. Add 100 μl of primary antibody and incubate for 2 h at room temperature.

12. Wash three times for 10 min with 200 μl 1x PBS at room temperature.

13. Tap plates on paper to remove excess liquid in-between washing steps.

14. Prepare a 1:50 000 dilution of secondary antibody (goat anti-rabbit IgG Alkaline Phosphatase-conjugated antibody).

15. Add 100 μl of secondary antibody and incubate for 2 h at room temperature

16. Wash three times for 10 min with 200 μl 1x PBS at room temperature

17. Tap plates on paper to remove excess liquid in-between washing steps.

18. Prepare p-nitrophenyl phosphate solution in dH_2_O and mix thoroughly until fully dissolved. Store at 37°C in the dark until used.

19. Add 100 μl of p-Nitrophenyl phosphate solution and incubate for 30 min at 37°C in the dark.

20. If necessary, reaction can be stopped by addition of 3 M sodium hydroxide.

21. Measure optical density at 400 nm in a spectrophotometer.

#### Quality control

##### Alizarin Red Staining

1. Prepare a 2% Alizarin Red Solution and mix thoroughly on a vortexer until completely dissolved.

2. Adjust pH to 4.2 by adding 0.5% ammonium hydroxide.^g^

3. Wash chitosan coated culture ware three times with 2 ml dH_2_O for 5 min on a shaker.

4. Add 2 ml of Alizarin Red solution and incubate for 5 min on a shaker at room temperature.

5. Carefully remove Alizarin Red Solution using a pipette.

6. Wash four times for 5 min with 2 ml dH_2_O on a shaker at room temperature.

7. Dry upside down over night.

8. Images can be taken in an inverted microscope equipped with a camera.

##### Crystal Violet Staining

1. Remove cell culture media.

2. Wash carefully with 2 ml 1x PBS.

3. Remove PBS and add 2 ml of 0.5% crystal violet solution.

4. Incubate for 30 min at room temperature without shaking.

5. Carefully remove crystal violet solution without disturbing the cell layer.

6. Wash four times with 2 ml 1x PBS.

7. Wash once with 2 ml tap water.

8. Dry upside down over night.

9. Images can be taken in an inverted microscope equipped with a camera.

## End notes

^a^Appropriate surface characteristics of tissue culture plastic used for coating with chitosan membranes is essential for the outcome of the experiment. We have tested three commercially available brands/surface treatments for tissue culture plastic, including “Primaria” (surface modified polystyrene; Falcon), “Nunclone” (Nunc) and non-tissue culture treated polystyrene plates (Falcon). Cell attachment on membranes prepared on these plates could not be sustained for more than a few days, whereas the identical procedure on the vacuum gas plasma treated plates allows for several weeks of cell attachment. Since the chitosan membranes prepared with this protocol are very thin, the surface of the tissue culture plastic can certainly affect elementary surface characteristics [22]. Furthermore, the differences in surface treatment could result in slight changes in the charge of the plate, thereby affecting the bonding of chitosan membranes to the plate surface.

^b^Since chitosan solution during the “low volume” preparation process is not directly stirred, but rather heated, a higher concentration of acetic acid is required for full dissolution. We have found that concentrations up to 10% of acetic acid do not affect bioactivity of MC3T3-E1 cells *in vitro*.

^c^The suitability of chitosan derivatives for solution casting of membranes can be assessed by their ability to completely dissolve during this step. Dispersion of particles, even after increasing the concentration of acetic acid to 10% -using either low or high volume preparation steps-, will result in sedimentation during the centrifugation step and finally in in-homogeneously cast membranes. Generally, in-homogeneously cast membranes do not sustain cell attachment. However, as long as the chitosan solution is clear, any differences in colour do not affect bioactivity.

^d^We have found that complete replacement of cell culture media every second day is critical to sustain cell attachment on chitosan membranes, especially with lower degree of deacetylation preparations. The use of DMEM/F12 media instead of the generally recommended αMEM media for this cell type, results in better bioactivity of chitosan membranes and increased cell attachment. Nevertheless, expansion of MC3T3-E1 cells before seeding on chitosan membranes should be performed in αMEM media for best growth behaviour.

^e^Since the volume of glutaraldehyde used for 0.02% cross-linking reactions is generally very low (~4 μl), preparing a 100 μl mixture in water will provide better distribution in the relatively high volume of chitosan solution. The small amount of 100 μl distilled water will not statistically affect the final concentration of the chitosan solution.

^f^Chitosan membranes cross-linked with 0.02% glutaraldehyde turn slightly yellow/orange based on the reaction between the primary amino group of chitosan and the aldehyde group of glutaraldehyde, resulting in the formation of an imine bond.

^g^A pH of 4.2 is recommended for the use of Alizarin Red to stain calcium deposits [24]. However, we have observed that Alizarin Red strongly stains chitosan membranes also in the pH range of 4.1 – 4.7.

## Abbreviations

αMEM: Minimum Essential medium alpha; BSA: Bovine serum albumin; DD: Degree of deacetylation; DMEM/F12: Dulbecco’s Modified Eagle Medium: Nutrient Mixture F-12; ELISA: Enzyme-linked immunosorbent assay; FBS: Fetal bovine serum; PBS: Phosphate buffered saline; UV: Ultraviolet.

## Competing interests

The authors declare that they have no competing interests.

## Authors’ contributions

RL participated in the conception and design, developed and optimized the methodology, acquired the data, analysed and interpreted the results, drafted the manuscript and gave final approval of the version to be published. MD performed pilot experiments and initial optimization of methodology. GO and OES participated in conception and design, revised the manuscript and approved the final version for publication.

## Authors’ information

RL is a post-doctoral fellow and group leader of research in tissue engineering and regenerative medicine at the department of Research and Development in the REModeL Lab, The Blood Bank, Landspitali University Hospital. MD is a biomaterial scientist working in the blood processing department of the Blood Bank, Landspitali University Hospital. GO is project manager in the department of Materials, Biotechnology and Energy at the Innovation Center Iceland. OES is the principal investigator and manager of the REModeL Lab at the Blood Bank, Landspitali University Hospital and assistant professor at the School of Science and Engineering, Reykjavik University.
